# Editorial: Advancing AI-driven code generation and synthesis: challenges, metrics, and ethical implications

**DOI:** 10.3389/frai.2026.1816684

**Published:** 2026-04-15

**Authors:** Sumeet Kaur Sehra, Sukhjit Singh Sehra, David S. Allison, Jaiteg Singh

**Affiliations:** 1 Conestoga College, Kitchener, ON, Canada; 2 Wilfrid Laurier University, Waterloo, ON, Canada; 3 Chitkara University Institute of Engineering and Technology, Chitkara University, Rajpura, Punjab, India

**Keywords:** artificial intelligence, challenges, code generation, ethical implications, synthesis

## 1 Introduction

Artificial intelligence (AI) has become an integral part of contemporary software engineering practice. Automated programming techniques, particularly AI-driven code generation, are increasingly used to accelerate development, reduce repetitive effort, and lower barriers to software creation. Recent advances in large language models (LLMs) have substantially expanded the scope of program synthesis, enabling systems that generate executable code directly from natural language descriptions (Jiang et al., 2026; Lyu et al., 2025). These capabilities have prompted widespread interest across research, industry, and education, positioning code generation as one of the most visible applications of generative AI.

Despite this progress, fundamental challenges remain. LLM-based systems often struggle with complex programming tasks that require precise interpretation of problem intent, multi-step reasoning, and iterative refinement (Ferrag et al., 2025). Also, there are persistent challenges related to correctness, evaluation, human trust, and ethical responsibility (Afroogh et al., 2024).

This research topic was motivated by the recognition that addressing these challenges requires more than continued improvements in model architecture or scale. Progress in AI-driven code generation depends equally on how systems are designed and orchestrated, how their outputs are evaluated, and how their use is situated within human and organizational contexts.

## 2 Overview of the contributing articles

The articles in this research topic collectively examine how AI-driven code generation can be advanced through improved system design, evaluation practices, and attention to broader implications. Although the contributions vary in scope and methodology, they share a common goal of moving beyond surface-level performance gains toward more reliable, interpretable, and context-aware code synthesis.

One contribution, *Blueprint2Code: a multi-agent pipeline for reliable code generation via blueprint planning and repair* (Mao et al.), presents a multi-agent framework that reframes code generation as a staged process encompassing task interpretation, explicit solution planning, implementation, and iterative repair. By decoupling planning from execution and incorporating test-informed debugging, this work demonstrates how system-level orchestration can mitigate common failure modes associated with single-pass generation and improve overall robustness.

Another article, *The Test Pyramid 2.0: AI-assisted testing across the pyramid* (Desai et al.), focuses on evaluation from the perspective of software testing. By extending the traditional test pyramid to reflect AI-assisted development workflows, this contribution highlights the limitations of narrow correctness-based metrics and argues for validation strategies that better capture maintainability, integration challenges, and longer-term system behavior.

A more exploratory contribution, *A quantum-inspired, biomimetic, and fractal framework for self-healing AI code generation: bridging responsible automation and emergent intelligence* (Nehzati), investigates self-healing approaches to code generation inspired by quantum systems, biological processes, and fractal structures. This work shifts attention toward adaptive and autonomously correcting architectures, raising important questions about controllability, transparency, and responsibility as automation becomes increasingly sophisticated.

The Research Topic also includes *AI-assisted design synthesis and human creativity in engineering education* (Tsakalerou et al.), which examines the role of AI-assisted code generation in human-centered settings. Focusing on engineering education, this contribution explores how generative tools influence creativity, learning, and design practices, emphasizing that code generation systems act not only as automation technologies but also as collaborators that shape problem-solving processes.

Taken together, these articles reflect a broader shift in the field toward treating AI-driven code generation as a structured, socio-technical process rather than a purely model-centric task. They highlight the growing importance of integrating architectural design, rigorous evaluation, and human considerations alongside continued advances in model capability.

## 3 Outlook and future directions

Collectively, the contributions in this research topic suggest that future progress in AI-driven code generation will depend on more than incremental gains in model performance. Advances in system architecture, evaluation methodology, and ethical awareness are increasingly central to building trustworthy, deployable code-generation systems. The works presented here point toward a more holistic research agenda in which technical innovation is coupled with careful consideration of context, responsibility, and real-world use.

By bringing together these perspectives, this topic aims to support ongoing dialogue at the intersection of machine learning, software engineering, and responsible AI, and to encourage future research that advances not only what code generation systems can produce, but also how and under what conditions they should be used.
